# Ethics of Gamification in Health and Fitness-Tracking

**DOI:** 10.3390/ijerph182111052

**Published:** 2021-10-21

**Authors:** Chirag Arora, Maryam Razavian

**Affiliations:** 1 Philosophy and Ethics Section, Eindhoven University of Technology, 5612AE Eindhoven, The Netherlands; 2 Information Systems Group, Eindhoven University of Technology, 5612AE Eindhoven, The Netherlands; m.razavian@tue.nl

**Keywords:** gamification, fitness tracking, ethical issue, gamification ethics, digital health

## Abstract

The use of game-like elements is become increasingly popular in the context of fitness and health apps. While such “gamified” apps hold great potential in motivating people to improve their health, they also come with a “darker side”. Recent work suggests that these gamified health apps raise a number of ethical challenges that, if left unaddressed, are not only morally problematic but also have adverse effects on user health and engagement with the apps. However, studies highlighting the ethical challenges of gamification have also met with criticism, indicating that they fall short of providing guidance to practitioners. In avoiding this mistake, this paper seeks to advance the goal of facilitating a practice-relevant guide for designers of gamified health apps to address ethical issues raised by use of such apps. More specifically, the paper seeks to achieve two major aims: (a) to propose a revised practice-relevant theoretical framework that outlines the responsibilities of the designers of gamified health apps, and (b) to provide a landscape of the various ethical issues related to gamified health apps based on a systematic literature review of the empirical literature investigating adverse effects of such apps.

## 1. Introduction

Gamification can be generally defined as the use of techniques and elements of video game design in non-game contexts [1,2]. In the context of health tracking and wearable health devices, gamification can be and is being used to encourage health and wellness activity. In particular, wearable activity trackers, in conjunction with gamified smartphone apps, have been promoted as promising tools for increasing physical activity among their users [3]. Some examples of game-like elements used in gamified health apps include points and rewards for health activity as well as social elements like competitions and challenges with other people [1,4]. Gamification is often distinguished from more immersive, full-fledged, or “serious games”, and the intention in gamification is to mimic experiences reminiscent of games to affect the behavior and motivation of users [5]. In the context of health, gamification generally seeks to alter user behavior by increasing their physical activity and/or adopting a healthier lifestyle through game-like experiences.

The use of such game-like elements, and gamification in general, is not unique to the case of health and fitness. Gamification techniques have found their application in a diverse range of areas including business organizations attempting to enhance customer engagement as well as employee performance, public policy initiatives, as well as in classrooms and other learning environments [6]. The increase in popularity of gamification in the past decade has been concurrent with the rise in accessibility of digital technologies, particularly smart phones as well as digital infrastructure that has created a networked world. Social networks, and other similar networked platforms, have also contributed to an increase in prevalence of gamification, as designers have leveraged such networks to improve interaction and engagement with users [5]. Despite its potential, gamification has also found its critics, including those who have questioned the moral and ethical legitimacy of gamification [2,7,8]. Kim & Werbach [2] have argued that such criticism suffers from painting with too broad a brush in denouncing almost all forms of gamification as vicious and/or exploitative. They also state that existing normative accounts of problems with gamification fall short of providing guidance to practitioners, particularly designers of gamified apps and platforms. Kim and Werbach have, instead, proposed a practice-relevant, context-sensitive and situated approach to the exploration of ethical issues associated with gamification. They have significantly enhanced the normative discussions about gamification, and to our knowledge, present the most comprehensive conceptual framework of ethical issues associated with gamification thus far available.

Yet, in their methodology, Kim and Werbach [2] were primarily concerned with business practices, and this may reflect the shortcomings of the study when applied to other contexts, such as health and fitness tracking. Further, while some recent studies have pointed out the “darker side” of health gamification, there is currently a lack of systematic reflection and compilation of such issues [9]. Recent studies also suggest that the negative effects of gamified health apps can have adverse effects on users motivations and lead to discontinuance of app use [9,10]. A thorough landscape of the ethical issues of gamified health apps could not only help designers carry out their potential moral duties towards the users of apps, but also lead to better long-term user engagement with their products. This paper seeks to advance the goal of facilitating a practice-relevant guide for designers of gamified health apps to address ethical issues raised by the use of such apps. More specifically, the paper seeks to achieve two major aims: (a) to propose a revised practice-relevant theoretical framework that outlines the responsibilities of the designers of gamified health apps, and (b) to provide a landscape of the various ethical issues related to gamified health apps as found in the empirical literature about such apps.

To achieve these objectives, we first conduct a theoretical analysis of the conceptual framework of ethical issues in gamification provided by Kim and Werbach [2]. The aim of this analysis is to propose amendments and refine the theoretical framework, in order to be useful particularly for the designers of gamified health apps. To this end, we created a tripartite framework based on the types of responsibilities designers of such apps may have. This tripartite theoretical framework can facilitate the taxonomizing of various ethical issues related to gamified health apps, based on how designers may address such issues. We then conducted a systematic literature review to investigate empirically supported ethical issues related to gamification in health and fitness tracking. The results of this review serve as a guiding list of ethical issues likely to be encountered in the design and use of a gamified health app. Such a review also allows us to explore the strength of our revised framework by investigating whether the kinds of responsibilities identified by our framework can address such issues. Finally, based on our analysis, we posit some limitations of this framework and offer suggestions for future studies that aim to locate further ethical issues related to gamified health apps or to test how such ethical issues actualize in specific circumstances or with particular gamified health apps. Our analysis also offers ways for users and, in particular, designers of such apps to navigate through, anticipate and avoid potential ethical issues related to gamified health apps.

## 2. Ethics of Gamification

The field of ethics is a branch within philosophy that deals with systemizing, defending and recommending concepts of right and wrong conduct [11]. As a response to social and technological developments, the twentieth century saw the development of an ethics of technology as a subdiscipline within ethics, which involved investigations specifically concerning the role of technology [12]. Gamification ethics, or the ethics of gamification, can be intellectually located within this tradition of the ethics of technology as part of a further sub-discipline—applied ethics—which seeks to apply theories, normative standards, concepts, and methods developed within ethics (or moral philosophy) to, for example, inquiries concerning specific technologies. Seeking to explore ethical issues raised by gamification, gamification ethics is a recent, yet rapidly developing topic [13].

Kim and Werbach [2] contend that prior to their work, gamification ethics displayed a tendency to over-generalize from particular examples and under-theorize, partly owing to the speed with which technologies associated with gamification advanced. To cover these gaps, they propose a “conceptual map of the terrain” that can offer normative guidance to gamification scholars as well as practitioners in identifying underlying structures that tie together what may seem like disjointed and disparate phenomena related to gamification. They share an aim with this paper: developing a framework that can be useful to the designers (and practitioners) of gamification. To this end, they propose four broad categories of ethical difficulties with gamification which encapsulate a cluster of concerns. In this section, we discuss these four categories as well as their underlying theoretical framework which allows these categories to be mapped onto a two-dimensional map. We then discuss the limitations of their framework and this conceptual map and propose a new framework that may help designers of gamified health apps locate the kind of responsibilities they may have to address ethical issues related to such apps.

### 2.1. Kim and Werbach Framework for Gamification Ethics

Kim and Werbach [2] Framework for Gamification Ethics. Kim and Werbach propose that the “ethical status of a practice of gamification, primarily, but not exhaustively, is determined by the extent to which the practice”:

1. Is exploitative;
2. Is manipulative;
3. Is intentionally or unintentionally harmful to the parties involved;
4. Has a socially unacceptable level of negative effect on the character of the parties involved.

Exploitation—Kim and Werbach argue that gamification is exploitative in situations where it is unfair to one party. For example, if, in the workplace, gamification techniques may benefit the employer by increasing employee efficiency, but these benefits may not be translated or trickle down to employees, or may be unfair to them in other ways (such as employees not being able to say no to such techniques), then gamification can be exploitative.

Manipulation—Kim and Werbach propose that since gamification essentially targets behavior change, it is prima facie open to the charge of being manipulative. In their discussion, they explore multiple accounts of manipulation, and offer two main ways in which gamification can be manipulative:

1. When the gamification elements and mechanisms are hidden from those it is applied on (deception);
2. When gamification techniques inhibit rational self-reflection and undermine autonomy in unjustifiable ways.

They state that it is largely an empirical question whether particular instances of lack of transparency of gamification techniques or the undermining of autonomy are manipulative. One may need more information, for example, to ascertain whether lack of transparency about game elements in a particular gamified health app is intended to deceive the user or not. As examples, they argue that addiction and distraction are two ways in which gamification can undermine autonomy.

Harm—Kim and Werbach [2] write that gamification can lead to both physical and psychological harm. Further, they state that, “*the risks of physical harm due to gamification primarily involve injury to others outside the gamified system, while the risks of psychological harms generally involve the players themselves*.”

Detrimental effects on character—One threat involved with gamification is that it can rely on rewards or incentives that are detrimental to one’s character. A standard example of the negative effects of an incentive to good behavior is a parent using candy to change or nudge their child’s behavior [2]. There are two related but distinct worries about gamification in relation to effects on character: (a) individuals relying on the wrong kinds of incentives, and (b) individuals excessively or obsessively relying on an incentive.

The analysis offered by Kim and Werbach relies on there being two primary reasons for these prima facie ethical issues related to gamification:

- Overlay of virtual and real norms;
- Conflict between the interests of individuals subjected to gamification and those who provide or design gamification elements.

Overlay of virtual and real norms—According to Kim and Werbach, gamification ethical issues such as manipulation or exploitation arise because gamification brings in conflict the different set of norms in play in “the real world” and the “game world”. For example, within a game, it may be acceptable to manipulate or deceive someone (such as in poker, for example). Yet, such a norm is hardly acceptable in the real world and if one were to apply a gamification technique that transposes a game-world norm to the real world, ethical issues may arise.

Individual vs gamification provider—The second source of ethical tensions, according to Kim and Werbach, is dissonance between motivations and interests of those subjected to gamification, and those who provide or deploy them. For example, in a gamified workplace, an employer may want to excessively track and reward employee productivity, but employees may consider this as an infringement of privacy.

This two-dimensional framework leads to the following conceptual map proposed by Kim and Werbach:

Here, they deem that exploitation and manipulation are “relational” concerns since they can only be evaluated in the context of the relation between individuals subjected to gamification and those providing/designing it. For example, as stated earlier, gamification, under this framework, is exploitative when there is an asymmetry or imbalance in the consequences of gamification such that the user either does not reap symmetrical rewards, relative to the designer, or even accrues harm. On the other hand, harms and detrimental effects to character can be evaluated “purely with reference to the players as individuals” [2]. Similarly, the dimensions of real-world and game lead to different relational and individual issues. Exploitation, according to this conceptual framework, is an issue where the gamification designer exploits a real-world vulnerability of the user, while manipulation is an issue that arises because the game elements are such that they inhibit a user’s autonomy. While this framework is helpful in understanding the four prima facie ethical issues related to gamification, there are also reasons to be skeptical about whether this framework is comprehensive or appropriate enough for locating ethical issues related to health gamification.

### 2.2. Theoretical Limitations of This Conceptual Framework

The two dimensions underlying the conceptual framework offered by Kim and Werbach—relational vs. individual and real-world vs. game-world—offer a good way to map several different ethical issues related to gamification. Yet, there are reasons to believe that the four categories defined by them—exploitation, manipulation, harm, and detrimental effects to character—only capture a narrow range of issues their conceptual framework has to offer. In what follows, we discuss the four categories further and, when applicable, present some limitations of applying these categories to the specific case of gamified health apps.

#### 2.2.1. Category 1: User-Designer Relation in the Real World, and Exploitation

Using the conceptual map (Table 1) offered by Kim and Werbach, the first category is one that should map the issues that relate to the relation between the designer and the users of a gamified system. Further, according to Kim and Werbach, these issues arise due to designers “exploiting a real-world” imbalance between designers and users [2]. There are at least two problems with labeling this category of issues as “exploitation”. First, it is not necessary that an imbalance between the designer and the user is a case of exploitation. A mere asymmetry in the distribution of rewards from the implementation of a gamified system does not constitute the wrong of exploitation. Second, there may be other kinds of wrongs that may arise out of the asymmetrical relationship between users and designers of a gamified system. Consider, for example, a gamified health app designed to motivate users to exercising more. In order to motivate its users, say that the app tracks a user’s activity, shares it with their friends on a social network, and gives them digital rewards if they outperform their friends. The social network, then, decides to allow third parties (such as other data brokers) to scrape this data off their network, which in turn, may lead to privacy harms to the user. It is hard to argue here that the designer is exploiting the user. Yet, the harm to the user is because of an imbalance between the designer and the user—namely, the choice of how the gamified app is designed and its data sharing policy rests with the designer and not the user.

This is not to argue that designers of a gamified health app cannot commit the wrong of exploitation. In the previous example, if the app itself was designed to scrape and store user data for sale to a third party, it may be deemed exploitative. The argument here is that exploitation is only one of the wrongs that may be involved in the category covered by the conceptual map (Table 1) offered by Kim and Werbach.

#### 2.2.2. Category 2: Game–User Relation in the Game World and Manipulation

Kim and Werbach term the second category of the ethical issues of gamification “manipulation”. This category, according to their conceptual map, tracks issues that can only be evaluated in the context of how a user interacts with the game elements. Kim and Werbach term them under “manipulation” as they arise because “providers have created an environment such that, in the game, the players cannot make autonomous choices, and instead make choices that serve the providers” [2]. Again, one problem in this phrasing is that it seems to exclude cases where users’ autonomy is undermined even without designers intending that to be the case or intending it for their own purposes. One could, for example, imagine a user of a gamified health app who is obsessively addicted to the game elements (such as in-game rewards like points or badges) even without the designer intending that to be the case. Kim and Werbach also cite addiction as an example of the kind of problem they have in mind here, and it is not sufficiently clear whether they want to restrict the category to cases of such addiction being a result of users “serving their (designers’) purpose”. Further, given the practice-relevant aim of the framework, the omission of such cases may not cover the full scope of the potential duties and responsibilities of designers and providers of a gamified system. One could argue that designers and providers of gamified systems are not merely responsible for consequences of intended actions, but also, at least some of the unintended actions. Many philosophers and ethicists, for example, believe that people should not only be held morally responsible for wrongdoings they are aware of, but also in cases where they should have known better [14]. Ascriptions of such moral responsibility (for should-have-known cases) may be even more justified in cases where the professional role of a person may morally require them to have known certain things. A doctor, for example, cannot claim ignorance for misdiagnosing a disease they were not, but should have been, aware of. In cases of gamified health apps, we may find similar cases where the designers and providers of such apps may be morally required to inquire into at least some of the ways in which the app undermines user autonomy.

Another limitation of discussions offered regarding both category 1 (user–designer in the real world) and category 2 (game–user relation) is that it does not distinguish between different roles providers and designers of a gamified system play. Kim and Werbach use both terms in their paper, but do not elaborate on how each could affect the system in their role. Distinguishing between the morally relevant actions available to each could be significant for the practice-relevant aims of the framework.

#### 2.2.3. Category 3: Harms to Individuals

The third category is not relational, in the sense that to evaluate ethical issues within this category, one need not look at the actions of the designer or the game elements. This category tracks a consequentialist approach to gamification ethics. A consequentialist approach to ethics, as the name suggests, is roughly the idea that whether an act is ethical or not depends on the consequences of that act [15]. Applied to the case of health gamification, this approach dictates that one only needs to look at the consequences of the game/gamified system, in the real world, to determine whether an individual has been harmed. In their introduction to this category, they state that it primarily involves physical harms to other individuals and psychological harms to the user of the gamified system. Yet, they do give some examples where the user may also be physically harmed, so the category should indeed include harm of a physical and psychological nature to both users of the game as well as others affected by it.

#### 2.2.4. Category 4: Detrimental Effects to Character

The fourth category deals with ethical issues that are also not relational and arise in the game. Kim and Werbach define this category as one which has issues that arise if *“there is an ethical lapse in the game, such that players act to satisfy the game’s objectives and are indifferent to fundamental human values”*. Yet, stating the problem this way also narrows down the potential problems involved here. Specifically, defined this way, the category leaves out issues where a user of a gamified system acquires character flaws that are not simply “lapses in the game” but also carry outside it.

### 2.3. Potential Problems Outside the Scope of the Framework

Besides the limitations already discussed, there may be additional problems that the framework does not address.

First, health, as a category, has an important social and structural dimension that may not be covered by a focus on individuals and their motivations. One’s health status, as well as one’s possibilities to engage in a healthy lifestyle, are conditioned by social factors such as one’s relative economic or social status. This may mean that the conflict between the motivations of individual players and gamification designers may not capture the entire breadth of ethical issues associated with gamification in healthcare. This may be further exacerbated by the fact that many gamified health apps also deliberately include social dimensions, such as leaderboards, competitions, badges, etc., and there is also evidence that users of such apps actively seek social validation in their gameplay [16].

Second, the dimensional contrast between the real world versus the game world may also be elusive. In their discussion of how to identify the relevant ethical concern for an individual, Kim and Werbach write:

“If the gamification activity produces an injury manifested in the real world, whether physically or psychically, the issue is one of harm. If instead there is an ethical lapse in the game, such that players act to satisfy the game’s objectives and are indifferent to fundamental human values, the issue is character.”

Yet, gamified health apps are not perfectly closed environments and there may be instances in health gamification where the game world may reinforce or affect the norms in the real world, and it may not be easily determinable whether the ethical concern arises in the game world or the real world. A gamified health app, for example, may not only push a player to satisfy the goals in the game, it may also change or influence what the player deems to be healthy in the real world too.

### 2.4. Framework for Designer Responsibilities

That there are limitations to the application of Kim and Werbach’s framework and conceptual map to the specific case of gamified health apps is not surprising. Kim and Werbach also anticipate this possibility, as they state that (a) their framework is conceptualized with the case of gamification in the workplace at the forefront, and (b) their attempt was not to provide a comprehensive mapping, leaving open the possibility of issues that may not be covered by their framework. Further, the discussion offered by Kim and Werbach does make important strides towards their practice-relevant aim of outlining ethical issues with gamification that could be useful for designers and providers of gamification. They also make the important observation that analyzing and identifying ethical problems with gamification requires more than just a consequentialist perspective in that not all the wrongs associated with such practices are related to the outcomes of the gamified system. Some of the wrongs, for example, are better analyzed from a virtue ethics approach, to figure out how a gamified system or app affects a user’s character. (In contrast to consequentialism, virtue ethics emphasizes moral character.) [17]. A virtue ethicist, for example, would recommend helping someone not for its consequences but because it is benevolent to do so. Similarly, from the point of view of the designers, which is the focus of this paper, a deontological perspective can give us crucial insights. Kim and Werbach, for example, state that in outlining the problems of exploitation and manipulation, they appeal to the deontological values of autonomy, fairness, and reason-responsiveness. In this section, we build on such insights offered by Kim and Werbach, and propose a new framework, geared towards outlining the types of responsibilities designers of gamified health apps have.

Before we outline our proposed framework, however, some important observations need to be stated. First, as discussed in Section 2.2, the designers of gamified apps may have the responsibility of preventing not just intentional wrongdoings, such as exploitation of vulnerabilities or undermining user autonomy, but also unintentional wrongdoings, especially cases where they can be expected to have known better. The latter may require designers, for example, to actively inquire into the outcomes of their designed apps, and as Kim and Werbach’s discussion illustrates, such inquiry should not be limited to a consequentialist perspective. Designers may also need to reflect on whether their design has negative effects on the character development of the users. Further, following a deontological (or Kantian) approach, designers may need to reflect on the possible wrongs that arise out of a designer’s lack of respect for the user or treating them as a mere means. On Kant’s view, we must always have respect for persons and there is something intrinsically wrong in treating them as mere means [18]. To treat them as mere means implies treating them only for our own ends and advantages, without regard for their interests [18]. For a designer to have respect for the users, and not treat them as mere means, implies being responsive to the needs and values of the users. Given that gamified health apps operate in the context of health, where special duties of care and beneficence are often emphasized, designers of such apps may even have special duties to actively consider the needs of the users [19]. In this sense, designers may be said to have design-related duties that are negative, in the sense that they require them to not harm the users, as well as duties that are positive, that require them to actively consider the good of the users.

Second, while designers have active responsibilities related to the design of the apps, other stakeholders may also share responsibilities for outcomes related to the use of an app. This includes users, but also providers, or other stakeholders who may force, push, or incentivize users to use such apps. One example could be a physician or a doctor who prescribes the use of a gamified health app to her patients. In such cases, these stakeholders may be more aware of the contexts within which a user is using the app, which may dictate that they also have moral responsibilities for outcomes for the users. Even in such cases, however, designers may also have responsibilities that are not limited to design. Such a model, where designers not only have responsibilities to make a “safe design” but also to actively and responsibly share responsibilities, has been argued by van de Poel and Robaey [20], amongst others. Under such a model, designers may, for example, be required to engage with such physicians and doctors in not only understanding the best design features, but also to communicate how to best integrate the app with other kinds of interventions physicians or doctors may be planning. Such communication-based duties may also be stated in terms of designers’ relation with, and as part of, the general society they inhabit. As stated, health as a category has an important social and structural dimension, and gamified health apps exist within such social and structural conditions. As far as possible, designers may need to engage with such social and structural systems to ensure that their apps are responsive to such conditions and that others also understand their role and utility as best as possible.

Another important stakeholder with whom designers may have to share responsibility is, of course, the user. From an ethical perspective, the need for such sharing or even transfer of responsibility to the user arises from the possibility that users may deviate significantly from what the designers intend or foresee as a way to use or engage with the gamified app. This includes misuse of the app in ways that are harmful to the user. One way in which designers can share or transfer responsibility to the users is through a “use plan” [21,22]. Summarizing the work of Houkes & Vermaas [22], Poel and Robaey [20] write that “the design of artifacts always includes the design of use plans, [where] a use plan is a sequence of actions with an artifact that will lead to the realization of a goal”. Such use plans may be communicated by the designer to the user through written manuals but also through other ways such as instructional videos, advertisements, etc., [20,22]. Further, use plans may even allow users to deviate from plans designers had in mind. Robaey [23] has argued that successful use plans should even consider such deviations, and encourage users to adapt to the use of artefacts in particular contexts to avoid hazards. To this end, Robae argues for epistemic access to the design of the artefacts, such that the artefact is not a black box for the user. The point here is not to argue that gamified health apps should necessarily have such use plans, but that designers of gamified health apps may have duties and responsibilities related to the successful transferring or sharing of responsibilities to/with the users of the apps.

With these observations in mind, we can now propose our framework, based on the three types of responsibility designers of gamified health apps may have:

1. Responsibilities for proper design—As the name suggests, this includes the responsibilities of the designers directly related to the design of their gamified health apps. This involves, for example, negative duties which require designing the game elements such that the users are not harmed or wronged, as well as potentially positive duties which help or facilitate the achievement of the user’s good. As stated, such duties may also involve designers actively inquiring into the consequences of their design activities.
2. Responsibilities to facilitate proper use—While design features are an essential part of facilitating an ethically good user experience, design and designers cannot account for all possible outcomes from the use of a gamified health app. There are various uncertainties and indeterminacies related to how users will, in practice, use the app. Avoiding wrongdoings because of, for example, misuse of the app, requires that designers share and transfer some of the responsibility to current as well as prospective users. As mentioned, one way to achieve this would be through use plans that designers can share with the users. There may also be other ways in which designers may encourage morally desirable behavior in users as well as foster the virtue of taking responsibility amongst users. One example may be through designer-organized forums and meetings that facilitate interaction amongst current and prospective users, such that they are able to share and create new beneficial ways of engaging with the apps that even designers may not have anticipated. There is evidence, for example, that such forums and meetings have helped members of the Quantified Self (QS) movement, which includes users of apps such as Fitbit, that measure and promote physical activity [24].
3. Responsibilities related to ensuring proper embedding of the apps within the larger social context—Besides users, designers may also need to share responsibilities with other stakeholders associated with gamified health apps. This may include the general public, but may especially include actors whose actions are directly related to gamified health apps. This includes, for example, and as stated earlier, doctors and physicians who may want to use such gamified health apps in planned interventions for their patient groups. It may also include insurance companies who may want to include data from gamified health apps and offer users monetary incentives to be more physically active in demonstrable ways. As informed stakeholders who may understand the nuanced ways in which the actions of actors such as the aforementioned insurance companies and physicians may affect users of gamified health apps, designers may have the responsibility to engage in interactions with other actors to facilitate the use of such apps in ways that promote better outcomes. The designers’ duties may also involve pushing forward and facilitating an active and democratic societal discourse on how such apps may be used and integrated within a given society’s health system. This may especially include engaging with other designers of such gamified health apps. More generally, there is a need for designers to reflect more broadly on the wider social and economic implications of their apps.

As stated, this paper has the dual aim of providing a practice-relevant theoretical framework to address ethical issues with gamified health apps as well as to provide a landscape of various ethical issues related to gamified health apps as found in the empirical literature about such apps. The first aim—the proposed theoretical framework—facilitates the taxonomizing of ethical issues related to gamified health apps, based on the type of designer action they may be addressed by. The second aim—of providing a landscape of empirically identified ethical issues related to gamified health apps—serves as a guiding list for designers of such apps and facilitates the addressal of such ethical issues. The attempt here is also to see how such empirically identified ethical issues may be addressed by the three types of designer responsibilities we have outlined here. Mapping the identified ethical issues on our practice-oriented framework would be an aid to the designers of gamified health apps who may seek to avoid harm to the users of such apps. In the next section, we discuss our methodology to answer the main question about what such effects on users of gamified health apps are: What ethical issues can be identified in the existing empirical work on the effects of gamification in health tracking?

## 3. Methodology

To answer our question, we conducted a systematic review of the literature on the effect of health gamification. We reviewed those publications that discuss the effects of gamified apps based on health and fitness tracking. The main aim of the systematic literature review, as stated earlier, was to achieve our second objective in this paper: facilitating a landscape of empirically identified ethical issues encountered in use of gamified health apps. While extracting these ethical issues from the empirical literature, we also noted recommendations for designers of gamified health apps given in the literature to address such ethical issues. We then mapped these recommendations onto our tripartite framework as proof of its utility in taxonomizing various ethical issues related to gamified health apps and corresponding designer responsibilities.

### 3.1. Protocol Overview

The study protocol consisted of the following steps:

1. Search for papers published after 2010 that discuss the effects of gamification in health and fitness apps (see Section 3.2 for details of search string and criteria).
2. Remove duplicates from the retrieved articles.
3. Apply the inclusion and exclusion criteria described in Section 3.2.
4. Apply backward snowballing method to systematic reviews within our reference list to find additional studies.
5. Check for sampling bias by searching for strings related to “ethics of health gamification”.
6. Extract data from the selected papers to answer our research question.

### 3.2. Search String, Strategy, and Database Selection

To select search databases and design our search string, we analyzed methodologies described in other systematic reviews on gamification in health (these are [5,25,26,27,28]. Since these reviews had different research questions than ours, we modified our search string and database list accordingly. Based on these reviews and the needs of our study, the electronic databases used included those identified as relevant to information technology, social science, ethics, psychology, and health: ACM digital library, Scopus, Web of Science, PubMed, PhilPapers, and IEEE explore. Following the account of the timeline of the popularity of gamification in health in [5,28], only papers after 2010 were included. While our main purpose was to identify potential ethical issues related to gamification in health and fitness, based on results and methodology used by [28], we were aware that such issues may be referred to in the literature as “negative”, “unintended” effects, “risks”, or similar terms, and designed our search string accordingly. Prior to applying the search protocol, we had also already identified that papers by [3,29,30] were relevant for our study. We, therefore, used these papers to use as a control, to make sure our search string did not skip relevant results. The following is our final search query (used for ACM database):

“query”: { Abstract:(gamif*) AND AllField:(health* OR medic* OR life* OR fitness OR well-being) AND AllField:(risk* OR danger* OR peril* OR effect* OR negative* OR unintended OR ethics OR ethical) }

“filter”: { Article Type: Research Article, Publication Date: (01/01/2010 TO 12/31/2020), ACM Content: DL, NOT VirtualContent: true }

This search strategy resulted in 621 results of which 459 were unique.

### 3.3. Screening and Selection of Papers

We then applied the following inclusion and exclusion criteria to narrow our search:

Inclusion criteria:

1. Peer-reviewed (incl. peer-reviewed conference papers);
2. Full papers (incl. full conference papers);
3. Clearly focused on gamification and described gamification elements (type of game design elements);
4. Addresses gamification in health and fitness tracking through use of devices and/or mobile apps;
5. Discusses empirical evidence related to the effects of such apps. The empirical evidence here denotes a reported effect of a gamified health app. The effect could be in terms of impact (affect, behavior, social, cognitive) or in terms of user experience when using the gamified health app.

The first two criteria were developed to maintain the quality of the articles. The second and last two were developed to make sure the literature clearly focuses on gamification within health and fitness tracking. We screened the articles initially based on their titles. We then consulted the abstract or the text of the article when it was necessary to reach a confident judgement. Based on these criteria, 80 relevant papers were found.

The exclusion criteria focus on excluding literature which only superficially mentions our terms of interest but does not contain sufficient detail for analysis:

1. Mentions health and fitness tracking but do not explicitly focus on gamification in such devices;
2. Addresses gamification in health tracking but does not give relevant empirical information on the effects of such gamification.

Here, relevant empirical evidence is deemed limited to:

1. Evidence about the effect of gamified health app on the user through qualitative user feedback (surveys, questionnaires, user reviews);
2. Evidence about potential negative effects of gamified health app through content analysis of the app.

In applying these exclusion criteria, the initially identified papers were carefully analyzed. Following this screening, we did backwards snowballing to two relevant systematic reviews, which also discussed the empirical effects of gamification in health, included in our list to retrieve additional papers. This gave us a list of 23 final papers. We also searched for multiple permutations of the strings “ethics of health gamification”, “negative effects of health gamification”, etc. in Google Scholar to check for papers published after 2010 that may have been missed. We manually screened through the first 50 results and did not find any relevant studies that had not already been included.

To facilitate objectivity, we piloted the process of inclusion and exclusion using 10 papers that were independently assessed by three different researchers, including the authors of this paper. The rest of the articles were screened for inclusion and exclusion after it was established that the three assessors agreed on the inclusion and exclusion assessment of the 10 articles.

### 3.4. Data Extraction and Analysis

All selected papers were read in their entirety, looking for relevant phrases, arguments, or discussion points that address some ethical issue or negative effects related to gamification in health and fitness tracking. For studies that were based on empirical evidence regarding subjective user experience of using a gamified health app, we only counted it as a reported effect and/or a related ethical issue when the study reported it as a significant effect, for example, because it was applicable for a significant number of users (and not, for example, when researchers expected to find it or mentioned it as a possible issue but which was not studied). Besides effects reported from such studies of user experience, we also included a couple of studies which were based on discourse analysis of gamified health apps. These studies looked at game elements and linguistic components (words used to describe health status or prospective users, for example) of the app and applied sociological theories to articulate the ethical issues at play with the apps. Through our analysis, we collected a list of all reported negative effects and/or related ethical issues of gamified health apps.

Of the selected papers, roughly 50% (12 out of 23) were based on qualitative studies and employed methods such as semi-structured interviews of a selected group of users of gamified health apps. These studies monitored the users over multiple days, ranging from one week to eight weeks. Nine (39%) were based on surveys or questionnaires conducted over a large number of existing users of gamified health apps. A third of the studies (33%) focused on a range of gamified health apps, while the rest were focused on a particular gamified health app. Among the latter, 33% (5 out of 15) used an app (or a prototype) not yet available in the public domain, while the rest employed use of an existing, often popular, app.

### 3.5. Results and Findings

Our review of the literature yielded various potential ethical issues with gamified health apps. Table 2 gives an overview of these issues along with sources citing such issues. While describing these issues, we also noted recommendations within the identified literature to designers of gamified health apps of ways in which they may potentially address these issues. We then mapped these recommendations onto our tripartite framework based on the type of designer responsibility a given ethical issue may be addressed by.

In Table 3, we encapsulate how the recommendations in the literature for designers of gamified health apps to address these ethical issues can be mapped onto our proposed categories. We indicate which type of designer responsibility may potentially address that particular ethical issue. It should be noted that while the table only includes recommendations in the literature, these are by no means an exhaustive set of recommendations to designers of gamified health apps related to addressing potential ethical issues. In the text below, as examples of further possible steps for designers, we also note some additional observations of our own. For example, although Maturo & Setiffi [30] analyze and introduce the issues of biosociality, amorality, and the neoliberal objection, they do not offer an explicit recommendation to address these issues. Their analysis, however, can be used to deduce some possible steps, and we note them below, in the text. Further, our recommendations are also not meant to be exhaustive and there are, of course, other steps designers could take to address some of these ethical issues. For example, we indicate that privacy-related issues may be addressed by proper design and proper use, as noted in the analysis presented above. This should not be taken to mean that there aren’t ways to address such privacy issues through means that may be characterized as belonging to the third category, of proper embedding in the social system. Table 3, and our analysis in general, indicate the scope for future research—particularly the need to investigate other ways in which designers of gamified health apps may address potential ethical issues by assuming one of the types of responsibility in our framework.

1. Privacy-related issues—Privacy was a chief concern among many users of gamified health apps. We found multiple studies that reported users being concerned about lack of privacy when using a gamified health app. This concern was either a result of users not comfortable with their data being tracked or shared, or because they were unsure how their data may be used by the app. Users also expressed concern with certain features of the app, intentionally designed, to lure them into using the app more or reminding them to use it. There was also evidence that some apps were intentionally designed to lure users into sharing more personal data [35]. There was also evidence of the privacy concerns of users translating into psychological concerns, such as feelings of being surveilled and corresponding anxiety. This clearly points to the need for designers to assume the responsibility of protecting user privacy. Orji et al. [31], for example, suggest that app designers should allow users to hide their identity and other personal information from other users of the app. They also suggest other “personalization” features to allow users to choose what information is shared and collected about them. Trang and Weiger [35] suggest that app providers should explicitly ask users’ permission before processing private information as well as inform users as much as possible about ways in which their information is used.
2. Cognitive manipulation—In their review of multiple gamified health apps, Maturo & Setiffi [30] write of apps exploiting concepts from cognitive psychology to manipulate users into using apps or oversharing information on them. Such design features are also partly responsible for the addicting nature of such apps, and Attig and Franke [3] have done an important study demonstrating the dependence of users on gamified health apps. Attig and Franke [3] write that such features rarely lead users into adopting an active lifestyle (or exercise) in the long run, and that designers should instead focus on facilitating the internal motivation of the users.
3. Dependence and addiction—Besides Attig and Franke [3], Barratt [29], in his qualitative study on the use of gamified apps by cyclists, also found evidence of such dependence and addiction to apps. Barratt also reported that some users also found their autonomy constrained, as they did not expect they would be so easily lured into the game rewards and incentives, such that they would complete the game challenges sometimes at the expense of other important personal and social commitments. At least some of these effects, at least to some extent, may be unforeseeable or unintended by the designers. It is hard to say from the available evidence the extent to which issues such as addiction or extreme dependence on an app are always solely a result of design features and not unhealthy ways of engaging with an app on the part of the user. As mentioned earlier, Attig and Franke write that app dependence rarely translates into user’s adopting a healthy lifestyle in the long run, and designers are better off aiming for the internal motivation of users. They suggest apps that allow for self-determination and are self-rewarding for users. Some of this may also be rectified by designers sharing or transferring responsibility (for proper use) to users. Yet, in so far as these issues are foreseeable, some, or significant responsibility also lies with the designers of apps, depending on the circumstances and game elements of the app.
4. Psychological harm—A similar case exists for design features that potentially lead to psychological harm to the users other than dependence or obsession with game rewards. These include, as stated earlier, feelings of being surveilled, and not feeling under control (lack of perceived autonomy). Some users also experienced extreme psychological states (such as anger or anxiety) because of gamified health app. This could be sometimes caused by lagging in the competition (or not having enough game rewards) or also when users suspected others of cheating [41]. Some design features seem to be responsible for incentivizing users to cheat, although part of the responsibility, again, lies with the users as well. A more concerning psychological aspect of gamified health apps seems to be their detrimental effects on existing internal motivations, as well as on the confidence of users [40], for example, point out that some users can be left with a strong sense of defeat, and it is therefore very important that game elements are designed to avoid such scenarios, particularly in serious contexts such as gamified systems for improving heart activity. There is definitely a case to be made for designers to review such cases and ensure that design features minimize the occurrence of such negative effects as much as possible. Besides the moral implications of such negative effects on users, evidence also suggests that it has adverse effects on user engagement with apps and leads to discontinuance [9]. Recommendations within the literature include: giving users more autonomy and personalization of app features [31], allowing cheating to a limited extent (for example, by allowing users more autonomy over how their results are displayed and building an app community that is tolerant of individual users making such choices in order to save “face”) [41], avoiding giving users a sense of defeat in serious apps [40]. Physical harms—Gamified health apps use game elements to motivate users to increase physical activity in their lives. However, for some users, this may result in side effects such that they may overexert themselves or engage with the app in ways that are harmful to them. The most obvious evidence of physical harm was through reports of users overtraining or overstressing themselves in search of game rewards [29]. At least some of these harms may be reduced through the use plans and other strategies designers may employ to transfer responsibility for proper use to the users. As discussed, there may be other ways of fostering virtuous use of apps amongst users by facilitating forums and other places where users may learn from each other how they can best engage with an app.
5. Hermeneutic problems—Designers and design features also seem to be directly responsible for various “hermeneutic” problems posed by gamified health apps. This problem relates to the use of terms within the app that may reinforce stereotypes. Lupton & Thomas [46], for example, write of gamified pregnancy apps which represent pregnant women in stereotypical ways, such as a Barbie doll.
  A related concern comes from Maturo and Setiffi [30] who argue that gamified health apps “atomistically insulate” individuals from other individuals even though, simultaneously, the individuals are “widely socially connected through a potential network of app users”. This insulation of users brackets out the social determinants/dimension of health in a sort of hermeneutic reductionism [30]. This hermeneutic reduction can lead to a phenomenon that Cheng [48] describes, where users feel pressured or compelled to look for and log only particular types of data, possibly at the cost of what they may have found meaningful or motivational. For example, Cheng [48] notes that “by only providing functionality to record performance metrics (i.e., distance, duration and location of a run), and rewarding based on these metrics, the Nike+ system implicitly communicates the other enjoyable aspects of running, such as the runner’s high, or the mindful interaction between human and environment, are less important”. Additionally, the proxies used in gamification elements can come to represent definite truths about what they are gamifying, as well as become privileged over other ways of knowing. This points to the problem of gamified health apps not properly embedded within a larger structural context.
6. Biosociality—The problem entails that certain gamified apps may reinforce physical stereotypes and also force the formation of groups based on such physical attributes [30]. Designers’ efforts of fostering and encourage virtuous behavior for proper use among users may partly address this problem.
7. The Neoliberal objection—As previously stated, factors such as education, income, and living condition have a huge influence on one’s health status [30]. Designers of gamified health apps should also be aware of the social dimensions and contexts within which their apps are used. It has been argued that the individualistic view underlying gamified health apps can lead to problems such as the depoliticization of the role of the state, which reduces the responsibilities of the state for the health of its citizens and shifts the burden to individuals. This objection states that such apps “foster a neoliberal ideology that implicitly stigmatizes people who are not capable of meeting the standard definition of ‘healthy’” [30]. Through a discourse analysis of major gamified health apps, Maturo and Setiffi [30] point out how the design and linguistic features of such apps may lead to such stigmatization. While designers can “fix” some of these linguistic issues, more a holistic solution to such problems perhaps lies in more active engagement of the designers with other actors and stakeholders in society. This could enable a more successful integration and embedding of gamified health apps within a larger structural quest to promote healthy lifestyle and outcomes for citizens.
8. Amorality—Another detrimental effect of gamified apps is that they may lead to/incentivize users to choose goals that are potentially harmful without caveats. This issue may be characterized as one of individual users choosing the wrong kind of incentive within a game, and it may be partly addressed by both better design features and virtuous user engagement with the app. Yet, as Maturo and Setiffi [30] point out, this can be more than an individual issue, and one where social norms may play a part. For example, dieting apps may lead a user to choose goals that other users are accomplishing or people around them find healthy, rather than what may actually be healthy for the individual.
9. Issues related to providers and facilitators in specific contexts—Finally, there is also evidence of there being merit in designers engaging with providers or facilitators of gamified health apps in particular contexts such as doctors and physicians. Writing about the use of gamified health app in the context of therapy (for mental well-being), [47], for example, write how therapists could benefit from having more control (and hence, responsibility) over the features of apps, and that this could be done through direct interactions between the designers of such apps and the therapist planning an intervention that uses the app.

**Table 3:** Recommendations in the literature to app designers to address ethical issues.

Reported Effect	Proper Design	Proper Use	Proper Embedding in the Social System
Privacy-related Issues	Personalization to allow users to choose what information they want to share Explicit notification and seeking user permission before processing private information	Inform users explicitly about how their private data will be used	-
Cognitive Manipulation	Avoiding manipulative features	Providing warning and safety restrictions against harmful cognitive effects	-
Dependence and Addiction	Allowing users to be more self-determined and self-rewarding in the usage of the app rather than offering extrinsic pre-determined rewards	Giving users more control over reward features	-
Psychological harms	Avoiding psychological harms such as a sense of defeat in serious apps Giving users more autonomy in choosing their goals Tolerating some level of “cheating” from users if that translates to better health choices	An empathetic approach to design that allows users to be autonomous and some self-determination over their goals as well as how they might be displayed	Facilitating a community of users who are empathetic to other users of the app
Physical Harms	Allowing users to choose their own goals	Improving user autonomy as well as giving warnings about dangers of overuse and exertion	-
Hermeneutic Problems	Avoiding game elements/rewards/terms that may reinforce harmful stereotypes	-	Avoiding reductionism in rewarding systems/game elements, for example, in ways that may incentivizes users to interpret their health and lifestyle in potentially harmful terms and/or though narrowly conceived metrics
Issues related to providers and facilitators	-	-	Engage with providers and facilitators of gamified health apps such that the apps cater to appropriate contextual information.

## 4. Discussion and Recommendations for Future Research

Our first task in this paper was to analyze and revise the framework offered by Kim and Werbach to identify ethical issues in gamification. Based on a theoretical analysis of this framework and arguments from moral theory, we argued for a revised practice-relevant theoretical framework that suggests three broad categories of responsibilities designers have in addressing ethical issues in gamified health apps. We have argued that the categorization, and the framework encapsulating this categorization we propose are better-equipped to help practitioners, such as designers, in the specific context of gamified health and fitness apps. This categorization also served as a guideline to identify and map ethical issues that have been discussed in the empirical literature on gamified health apps. We presented these in Table 3 in Section 3. We want to emphasize that, theoretically, there are more issues as well as steps designers could take to address those issues, within the 3 categories in Table 3, that we did not find in the empirical literature on gamified health apps. While this may partly be because of the limitations of design and methodology of our study, it also points to a space for further research that looks for evidential proof of other issues (and corresponding steps to address them) that one can anticipate based on other literature on games and gamification. For example, there is a possibility that gamified health apps may lead to a trivialization of health as an unintended effect of game elements that try to simplify complex health variables for users of an app. Nguyen ([49] has theorized a similar possibility, arguing that one possible effect of gamified health apps might be a simplification of users’ health goals. For example, a user may get so obsessed with numbers or rewards on their gamified health app that they may lose track of their original goal of being healthy. Similarly, one may possibly observe ethically problematic effects, other than those we found in the empirical literature, because of the use of gamified apps. Zuboff [50], for example, has argued that many digital environments, including health-related apps, commodify user behavior in the interests of the designers of these environments. Our review indicates the possibility for empirical investigation of such a hypothesis in the context of gamified health apps in future work.

It should be noted that our attempt in this paper was to locate as many ethical issues related to gamified health apps as we could find in the empirical literature. One limitation to note here is that there are related areas of research, such as research focusing on behavior change technologies in general, which also deal with some similar ethical issues. Future research may seek to broaden the scope of our research to include ethical issues identified from those domains as well. Another related limitation is that some of the ethical issues may need more or stronger evidence, particularly about the extent to which they are universally or even widely operational across gamified health apps. For example, we stated that Maturo and Setifi’s [30] work on gamified health apps, which is based on analysis of the design features of the apps, as well as a discourse analysis of reviews of the apps on various internet forums, notes that such apps may lead to stigmatization of people who may not be able to meet standard definitions of “healthy”. Yet, it is also a possibility that, in practice, user communities (facilitated by forums and groups, for example) may subvert the affordances of such apps, and negate such tendencies of stigmatization. The evidence for such optimism comes from other works on self-tracking (not necessarily gamified) health apps. Sharon & Zandbergen [51], for example, through their study of self-tracking communities, elucidate how theoretically postulated ideas about self-trackers, such as their engagement in a form of “data-fetishism”, are limiting. They assert that instead of being obsessed with narrow notions of objectivity (an idea encapsulated within the data-fetishism charge against self-trackers), self-tracking communities actually attribute meaning to their quantified data in ways that resist such objectivity. Further, self-trackers also use their practice to resist social norms (instead of reinforcing them) as well as invent imaginative ways of using self-tracking as a narrative aid [51]. Their study points to the need for further ethnographic and anthropological studies of self-trackers, as well as users of gamified health apps, to understand how such users and communities of users may resist theoretically anticipated problems with such apps. This is not to say that the theoretically postulated and anticipated sources of ethical issues with gamified health apps are not of value. Even if they are eventually resisted by users of such apps, theoretical critiques may offer themselves as a source of critical reflection on behalf of the users as well as designers of such apps. Such users and designers may then use the analysis offered in these theoretical critiques to find ways of resisting and escaping the anticipated problems. This reiterates the need for the sharing of responsibilities between the various stakeholders related with gamified health apps.

This brings us to a final point about the utility of the analysis offered in this paper. Our aim has been to provide a practice-relevant framework to identify different types of designer responsibilities that can address ethical issues in gamification. Further, we also aimed at providing a landscape of various ethical issues related to gamified health apps as found in the empirical literature about such apps. This framework, based on designer responsibility as well as the list of various issues, can be useful for designers, users of gamified health apps, as well as other stakeholders, to anticipate as well as avoid ethical issues in their interaction with such apps. Given the recent evidence suggesting that ethical issues, such as potential psychological harm to app users [9], can lead to app discontinuance, addressing such issues may also serve to improve the long-term user engagement with such products. The designers of such apps, in particular, can use our framework to foresee possible issues as well as plan validation studies to ensure that the apps they design are able to avoid foreseeable ethical problems as well as problems that may arise as unintended and unforeseeable effects of their design features. The designer duties prescribed by our framework also emphasize the need for designers to reflect more broadly on the socio-economic implications of the technologies they seek to introduce and point to the potential utility of seeking more democratized approaches towards technological design.

## Figures and Tables

**Table 1:** Conceptual Mapping of Gamification Ethics.

Category	Real World	Game
Relational	Exploitation	Manipulation
Individual	Harm	Character

**Table 2:** Reported Ethical Issues in Gamified Health and Fitness Apps.

Reported Ethical Issue	Sources
Privacy-related Issues	[30,31,32,33,34,35]
Cognitive Manipulation	[30]
Dependence and Addiction	[3,9,29,33,36,37]
Psychological Harms	[10,29,31,37,38,39,40,41,42,43,44]
The Neoliberal Objection	[30]
Physical Harms	[29,45]
Hermeneutic Problems	[30,46]]
Biosociality	[30]
Amorality	[30]
Issues related to providers and facilitators	[47]
